# Genome-wide survey of Calcium-Dependent Protein Kinases (CPKs) in five *Brassica* species and identification of CPKs induced by *Plasmodiophora brassicae* in *B. rapa, B. oleracea, and B. napus*

**DOI:** 10.3389/fpls.2022.1067723

**Published:** 2022-11-21

**Authors:** Junxing Lu, Nan Yang, Yangyi Zhu, Zhongxin Chai, Tao Zhang, Wei Li

**Affiliations:** ^1^ Chongqing Key Laboratory of Molecular Biology of Plant Environmental Adaptations, College of Life Science, Chongqing Normal University, Chongqing, China; ^2^ Wuxi Fisheries College, Nanjing Agricultural University, Jiangsu, China; ^3^ Department of Botany, University of British Columbia, Vancouver, BC, Canada

**Keywords:** *Brassica* species, calcium-dependent protein kinase, evolution, expression pattern, *Plasmodiophora brassicae*

## Abstract

Calcium-dependent protein kinase (CPK) is a class of Ser/Thr protein kinase that exists in plants and some protozoa, possessing Ca^2+^ sensing functions and kinase activity. To better reveal the roles that *Brassica* CPKs played during plant response to stresses, five *Brassica* species, namely *Brassica rapa* (*B. rapa*)*, Brassica nigra* (*B. nigra*)*, Brassica oleracea* (*B. oleracea*)*, Brassica juncea* (*B. juncea*)*, and Brassica napus* (*B. napus*) were selected and analyzed. In total, 51 *BraCPK*, 56 *BniCPK*, 56 *BolCPK*, 88 *BjuCPK*, and 107 *BnaCPK* genes were identified genome wide and phylogenetics, chromosomal mapping, collinearity, promoter analysis, and biological stress analysis were conducted. The results showed that a typical *CPK* gene was constituted by a long exon and tandem short exons. They were unevenly distributed on most chromosomes except chromosome A08 in *B. napus* and *B. rapa*, and almost all *CPK* genes were located on regions of high gene density as non-tandem form. The promoter regions of *BraCPKs*, *BolCPKs*, and *BnaCPKs* possessed at least three types of cis-elements, among which the abscisic acid responsive-related accounted for the largest proportion. In the phylogenetic tree, CPKs were clustered into four primary groups, among which group I contained the most *CPK* genes while group IV contained the fewest. Some clades, like *AT5G23580.1*(*CPK12*) and *AT2G31500.1* (*CPK24*) contained much more gene members than others, indicating a possibility that gene expansion occurred during evolution. Furthermore, 4 *BraCPKs*, 14 *BolCPKs*, and 31 *BnaCPKs* involved in the *Plasmodiophora brassicae* (*P. brassicae*) defense response in resistant (R) or susceptible (S) materials were derived from online databases, leading to the discovery that some R-specific induced *CPKs*, such as *BnaC02g08720D*, *BnaA03g03800D*, and *BolC04g018270.2J.m1* might be ideal candidate genes for *P. brassicae* resistant research. Overall, these results provide valuable information for research on the function and evolution of CDK genes.

## Introduction

Ca^2+^ is a generic form of the second messenger to participate in numerous signaling pathways (Hetherington and Brownlee, 2004; Zhang et al., 2014b). The change of Ca^2+^ concentration in cells can be captured by calcium sensors. Based on whether or not they are adjusted by the combination with Ca^2+^, calcium sensors are divided into four classes in plants, namely calmodulin (CaM) or CaM-like proteins (CMLs), calcineurin B-like proteins (CBLs), CBL interacting protein kinases (CIPKs), and the calcium-dependent protein kinases (CPKs) and their relatives, CPK-related kinases (CRKs) (Luan et al., 2002; Yang et al., 2021). Among them, CPKs exist in plants and some protozoa, especially in plant extracts. Additionally, rich calcium-stimulated protein kinase activity is found to be related to *CPKs* (Harmon et al., 2000). Typical CPK proteins contain an N-terminal variable domain, a protein kinase domain, an autoinhibitory domain, and a calmodulin-like domain (Cheng et al., 2002). The protein kinase domain is the catalytic domain containing an adenosine triphosphate binding site adjacent to the inhibitory domain, and the successive calmodulin domains always contain a protein kinase domain and EF-hands to determine whether the CPK is calmodulin dependent (Cheng et al., 2002; Hrabak et al., 2003).

Numerous studies have reported that CPKs are involved in plant responses to abiotic stresses. For example, *Arabidopsis CPK1* and *CPK2* were rapidly expressed under drought and high salinity stress, and *CPKs* played a significant role in enhancing abiotic stress tolerance (Urao et al., 1994). In maize, 12 of 40 CPK genes responded to low temperature, drought, salt, ABA, and hydrogen peroxide (Kong et al., 2013). In wheat, 10 of 14 *CPK* genes were responsive to abiotic stress, including drought, NaCl, and ABA. In potatoes, *StCPKs* were responsive to biotic and abiotic stresses as well as hormone stimulation (Bi et al., 2021). Among them, 20 *StCPKs* showed differential expression patterns in drought-tolerant and drought-sensitive potato varieties under drought stress, which allowed for recognition of abiotic stress of potatoes. In grapes, the response of *VpCPKs* to NaCl treatment and transcriptional responses at low and high temperatures has been studied (Zhang et al., 2015). Almost all 19 CPKs were up or down-regulated under at least one abiotic stress, but some genes had a rather mild response, and some grape *CPK* genes, like *VpCPK6, VpCPK9, VpCPK14, VpCPK16*, and *VpCPK19* had positive effects in fighting against powdery mildew. Overall, most CPK-related studies focused on the role in abiotic stress and rarely mentioned biotic stressors.

*Brassica* is a member of the *Brassicaceae* family, which contains many important vegetables, oil crops, and forage crops and has high economic value. *CPK* gene is a candidate gene for modifying plant stress resistance cations. Although there are some studies on CPKs of *Brassica* plants, such as turnip and canola (Zhang et al., 2014a; Wang et al., 2017), there are few studies on *Brassica* CPKs as a whole. Additionally, most of the studies on *CPKs* are conducted in one type of plant, which cannot be accurately used for comparison between plants. In this study, five *Brassica* Species (*B. rapa, B. nigra, B. oleracea, B. juncea, and B. napus*) *CPK*s (*BraCPKs, BniBCPKs, BolCPKs, BjuCPKs, BnaCPKs*) were identified, for phylogenetic, chromosomal mapping, collinearity, promoter analysis, and biological stress analysis. This study specifically studied the *Brassica* CPK induced by clubroot, which is caused by the obligate biotrophic pathogen *P. brassicae*, one of the most devastating diseases affecting *Brassicae* (Zhang et al., 2016). Our results provide important information on the evolutionary history and biological function of the *Brassica* Species CPKs in biotic stress.

## Materials and methods

### Identification of *CPK* genes

To identify *CPK* genes in *Brassica*, the whole-genomes of five *Brassica* species were downloaded from the Brassica Database (BRAD) (Chen et al., 2022) (http://brassicadb.cn/). The HMMER profiles of the protein kinase domains (PF00069) and EF-hand domains (PF00036, PF13499, or PF13833) derived from Pfam (www.ebi.ac.uk/interpro) were used as queries to search for CPKs in the *Brassica* protein database, thereby obtaining the first part of the candidate CPK proteins. Furthermore, all *Arabidopsis* CPK proteins were obtained from TAIR (https://www.arabidopsis.org/) and used as queries to search orthologs in five *Brassica* species using the BLAST search analysis. Subsequently, the two sets of candidates were merged and subjected to NCBI-BLASTP using the UniProtKB/SwissoPrpt database, and their annotations were downloaded for further screening. Then, we excluded the sequences that were CPK-related proteins, calcium/calmodulin-dependent proteins, and calcium and calcium/calmodulin-dependent protein kinases. The remaining proteins were regarded as CPKs. The conserved domains and EF-hand quantity were predicted by SMART (Letunic and Bork, 2018; Letunic et al., 2021) (http://smart.embl-heidelberg.de/). N-terminal myristoylation sites, N-terminal palmitoylation sites, and N-terminal acetylation sites were predicted using NMT (https://mendel.imp.ac.at/myristate/SUPLpredictor.htm), CSS-Palm4.0 (Ren et al., 2008; Chica, 2020) (http://csspalm.biocuckoo.org/index.php), and NetAcet-1.0 (Kiemer et al., 2005) (https://services.healthtech.dtu.dk/service.php?NetAcet-1.0), respectively.

### Gene structure, conserved motif, promoter element, and phylogenetic analysis

TBtools (Chen et al., 2020) was used to visualize the exon-intron organization of *CPK* genes according to the annotation information of the *Brassica* genomes in BRAD. MEME suit (https://meme-suite.org/meme/index.html) was used to search for conserved motifs in CPK proteins (Bailey et al., 2015). Approximately 2,000-bp upstream flanking fragments of the CPK genes were derived from the genome, and PlantCARE (http://bioinformatics.psb.ugent.be/webtools/plantcare/html/) was used to predict promoter *cis*-elements (Lescot et al., 2002). Full-length *Brassica* species CPK protein sequences were aligned *via* the MUSCLE program and then used to construct a phylogenic tree *via* the neighbor-joining method of MEGA11.0 software (Tamura et al., 2021). Bootstrap values were estimated to assess the relative support for each branch with 2,000 replicates.

### Genomic distribution and synteny analysis

*Brassica CPKs* were mapped on chromosomes according to genome annotation files from BRAD. The segmental and tandem duplication regions were obtained using the MCscanX software (Wang et al., 2012). For synteny analysis, synteny blocks of the CPK genes were visualized using TBtools (Chen et al., 2020). Nonsynonymous (Ka)/synonymous (Ks) calculation analysis was performed by KaKs_calculator 3.0 (Zhang, 2022).

### Expression characteristics of *CPK* genes induced by *Plasmodiophora brassicae*

To study the CPKs induced by *P. brassicae* infection, three RNA-seq datasets of clubroot-resistant (R) and clubroot-susceptible (S) lines of *B.rapa* (PRJNA298858), *B.oleracea* (PRJNA298858), and *B.napus* (PRJNA345072) were downloaded from NCBI (Chen et al., 2015; Zhang et al., 2016; Li et al., 2019). The time points for *B. rapa* dataset were 0, 12, 72, and 96 hours post-inoculation (hpi). The time points for *B.olerascea* were 0, 7, and 14 days post-inoculation (dpi), and for *B.napus* were 0, 12, 24, 60, and 96 hpi. The TPM (Transcripts Per Kilobase Million) value was used to display the gene expression levels, and the heat map was drawn by TBtools (Chen et al., 2020). Correlation analysis was carried out according to their expression levels in the relevant time after *P.brassicae* infection, so as to judge the influence of *P. brassicae* in *Brassica* species.

## Results

### Identification and characterization of CPKs in *Brassica*

To identify CPKs in *Brassica*, we performed a genome-wide analysis of the *CPK* gene family in the five *Brassica* species through a BLAST search and HMMER analysis. Proteins with protein kinase domain and EF-hand domain were identified, and those that might possess similar domains, such as CPK-related proteins, calcium/calmodulin-dependent proteins, and calcium and calcium/calmodulin-dependent protein kinases were carefully excluded. Altogether, we identified 51 *BraCPKs*, 56 *BniCPKs*, 56 *BolCPKs*, 88 *BjuCPKs*, and 107 *BnaCPKs* from *B. rapa, B. nigra, B. oleracea, B. juncea*, and *B. napus*, respectively. The typical *CPK* genes were constituted by a long exon and tandem short exons (**Table S1**; **Figure S1**). Three quarters of *CPK* genes in five species possessed six to eight exons, with detailed proportions of each species possessing this number of exons at 80.39% (*B. rapa*), 78.57% (*B. nigra*), 78.57% (*B. oleracea*), 80.68% (*B. juncea*), and 73.83% (*B. napus*). The exon number ranged from 2 to 21, such as *BjuO012843* having only two short exons and *BraA02g042460.3C* exhibiting 21 exons of varying lengths.

In general, CPK proteins contained one Protein kinase and four EF-hand domains, with some CPKs varying in the number of EF-hand domains, ranging from one to six. (**Table S1**; **Figure S2**). Furthermore, the majority of CPK proteins possessed palmitoylation sites at their N-termini (*B. rapa*, 75.44%; *B. nigra*, 76.79%; *B. oleracea*, 73.21%; *B. juncea*, 69.32%; *B. napus*, 70.09%). This is crucial for regulation, as palmitoylation plays important roles in modulating proteins’ trafficking, stability and sorting (Martín and Busconi, 2000; Draper et al., 2007; Greaves and Chamberlain, 2007; Linder and Deschenes, 2007). Moreover, half of CPK proteins contained myristoylation sites, and several contained N-terminal acetylation sites.

### Chromosomal localization and gene expansion

**Figure 1:**
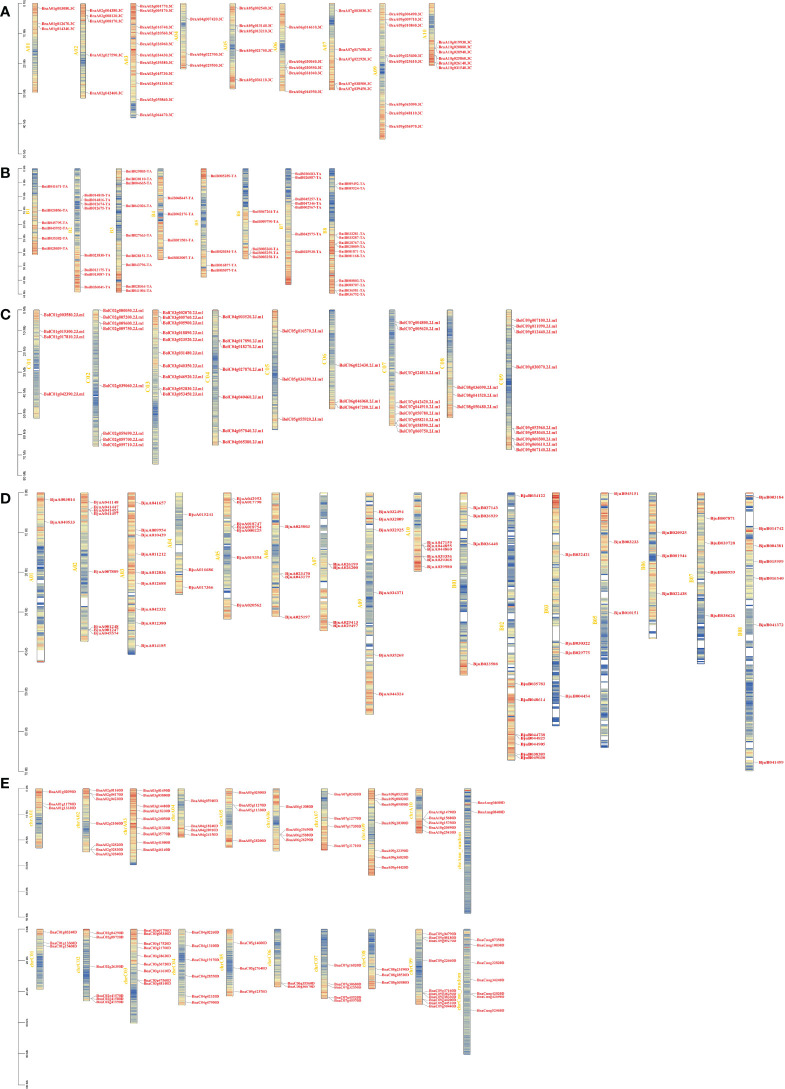
Locations of CPK genes on chromosomes in five *Brassica* species. Locations of CPK genes in *B rapa* **(A)**, *B*. *nigra*. **(B)**, *B*. *oleracea* **(C)**, *B*. *juncea* **(D)**, and *B*. *napus* **(E)**, respectively. A01-A10, B1-B8, C01-C09, A01-B08, and chrA01-chrCnn represent the chromosomes in *B rapa*, *B*. *nigra*, *B*. *oleracea*, *B*. *juncea*, and *B*. *napus*, respectively. Red indicates high gene density, while blue indicates low.

To understand the genomic distribution of *CPKs*, gene positions were acquired from the genome database of *Brassica* species (**Figure 1**). *BraCPK* genes were located on 9 out of 10 chromosomes with an exception of Chromosome A08 (**Figure 1A**). Chromosome A03 carried 11 evenly distributed *BraCPK* genes, which was the maximum number on one chromosome. Meanwhile, chromosomes A01 and A04 possessed only three *BraCPK* genes. 55 *BniCPK* genes were spread out across eight chromosomes, and among them, 12 *BniCPK* genes were located on Chromosome B08, mostly in cluster manner (**Figure 1B**). The *BniCPK* gene number on other chromosomes varied, ranging from four to nine. As seen in **Figure 1C**, *BolCPK* genes distributed on all chromosomes, and most genes clustered on chromosome C03 which was followed by chromosome C07, C09, and C02. As shown in **Figure 1D**, *BjuCPK* genes were unevenly dispersed on chromosomes excluding A08 and B04 which carried no *BjuCPK* genes. The gene number on different chromosomes varied greatly. For example, there are nine genes on chromosome A03 and only 2 genes on chromosome A01. Similar to the distribution of *BraCPKs* and *BolCPKs*, *BnaCPK* genes were unevenly dispersed on chromosomes. This uneven distribution excluded ChrA08 which was the only one that carried no *BnaCPK* genes (**Figure 1E**). The quantity of *BnaCPK* genes on different chromosomes ranged from 2 to 10. ChrC09 had 10 *BnaCPK* genes which was the largest number found on a single chromosome, while ChrC06 contained only 2 *BnaCPKs*.

Considering that *B. napus* is derived from the recombination of *B. rapa* and *B. oleracea*, *B. juncea* is derived from *B. rapa* and *B. nigra*, the positions of *BnaCPK* genes on chromosomes ChrAs or ChrCs and *BjuCPK* genes on chromosomes ChrAs or ChrBs showed highly consistent with those of corresponding homologous *BraCPK*, *BolCPK* or *BniCPK* genes. Additionally, almost all *CPK* genes were located on regions of high gene density as non-tandem form. In total, only seven *CPK* gene clusters were found in five species, including one in *B. rapa* (*BraA09g325600.3C* and *BraA09g325610.3C*), two in *B. nigra* (*BniB012674-TA* and *BniB012675-TA*; *BniB003258-TA*, *BniB003259-TA* and *BniB003260-TA*), one in *B. oleracea* (*BolC02g059690.2J.m1*, *BolC02g059700.2J.m1* and *BolC02g059710.2J.m1*), two in *B. juncea* (*BjuA001247* and *BjuA001248*, *BjuA026199* and *BjuA026200*), and one in *B. napus* (*BnaC02g41570*, *BnaC02g41580* and *BnaC02g415790*).

**Figure 2:**
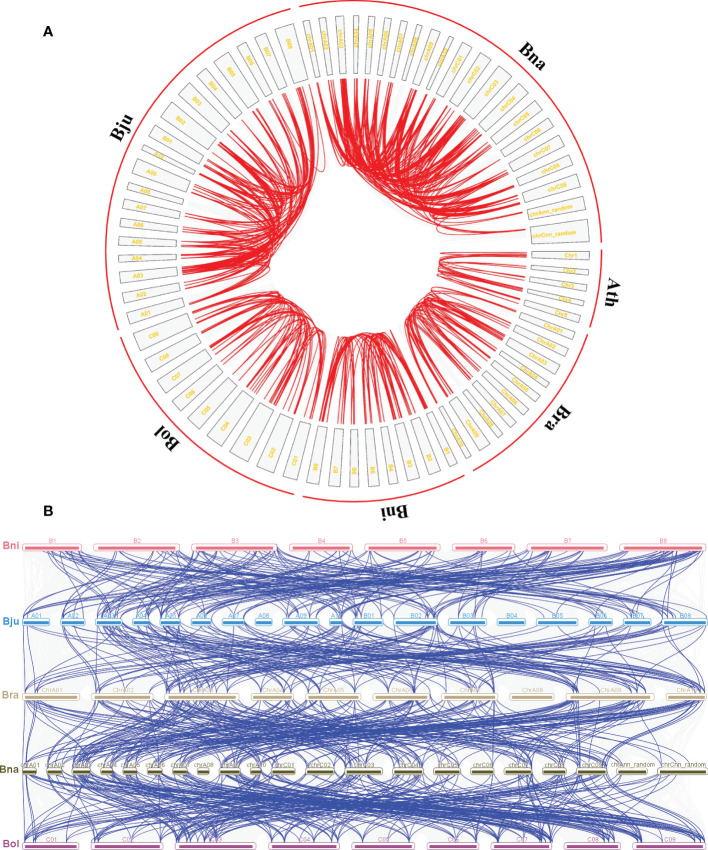
Duplication and syntenic relationship of CPK genes in five *Brassica* species. **(A)** Duplication of RLP genes in *A. thaliana, B. rapa, B. nigra, B. oleracea, B. juncea*, and *B. napus*, respectively. The red line represents the gene pairs and the gray box represents the chromosome. **(B)** Syntenic relationship of CPK-encoding genes between *B.rapa, B.nigra, B.oleracea, B.juncea*, and *B.napus*. The blue line represents the gene pairs and the colored box represents the chromosome.

To better reveal the expansion of *CPK* genes in five *Brassica* genomes, the duplication patterns of *CPK* genes were predicted within and between each genome with the *A. thaliana* genome added for comparison (**Figure 2**). The result showed that there were 15 *CPK* gene pairs in *A. thaliana*, and the gene pair number of *Brassica* species was tripled or even tenfold, namely, 47 pairs in *B. rapa*, 51 pairs in *B. nigra*, 52 pairs in *B. oleracea*, 154 pairs in *B. juncea*, and 200 pairs in *B. napus* (**Figure 2A**). Moreover, the possible syntenic relationship of *CPK* genes between *Brassica* genomes was also investigated. Subsequently, we obtained 234 orthologous gene pairs between *B. rapa* and *B. napus*, 177 between *B. rapa* and *B. juncea*, 258 between *B. oleracea* and *B. napus*, and 182 between *B. nigra* and *B. juncea*, which are shown in **Figure 2B**. Chromosome A03/A09, B03/B08, and C03/C07/C09 possessed the higher orthologous regions in *B. rapa, B. nigra* and *B. oleracea*, respectively. Correspondingly, chrA03, chrA09, chrC03, and chrC09 subgenomes were classified as higher orthologous regions in *B.napus*. These higher orthologous regions were not present in chromosome A03, A09, B03, and B08 subgenomes in *B.juncea*, which may be due to the imperfect assembly of the *B.juncea* genome.

Additionally, the nonsynonymous/synonymous mutations (Ka/Ks) analysis was performed on orthologous *CPK* genes between *A. thaliana* and the other five *Brassica species* (**Table S2**). The Ka/Ks value of the predicted *CPK* paralogs that had average values of 0.087 (*B. rapa*), 0.091 (*B. nigra*), 0.083 (*B. oleracea*), 0.088 (*B. juncea*) and 0.083 (*B. napus*) were less than one, suggesting that all pairs of CPK proteins were under strong purifying selection pressure.

### Phylogenetic relationships of *CPK*s

**Figure 3:**
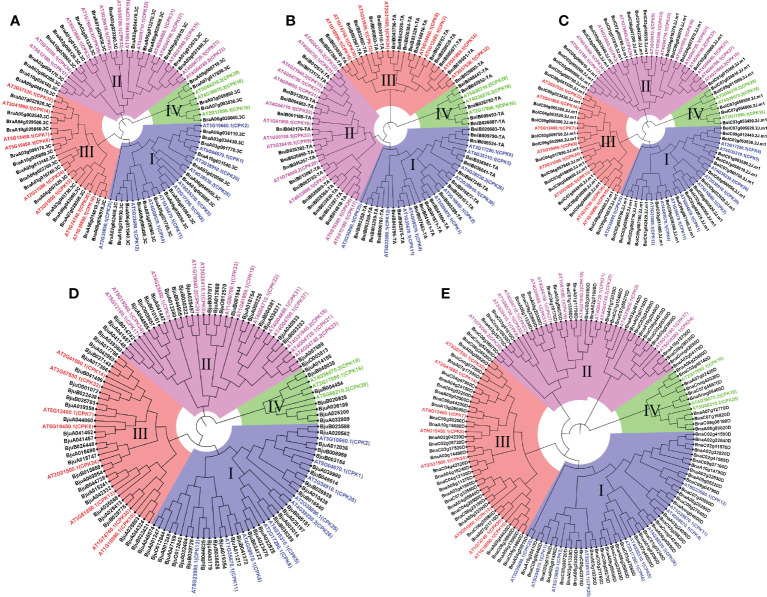
Phylogenetic trees of CPK proteins in five *Brassica* species. Phylogenetic trees of CPK proteins in *A. thaliana* and *B. rapa* **(A)**, *B. nigra* **(B)**, *B. oleracea* **(C)**, *B. juncea* **(D)**, *B. napus* **(E)**, respectively. The phylogenetic trees were generated by the NJ method with bootstrap analysis (2,000 bootstrap replicates) using an amino acid sequence alignment of CPK proteins from *A. thaliana* and *Brassica* species by the MEGA 11.0 program. The CPK proteins in each *Brassica* species were clustered into four groups (group I, group I, group III, and group IV).

To explore the phylogenetic relationship of *CPK* genes, neighbor-joining trees were constructed by CPK protein sequence from five *Brassica* species (**Figure 3**). CPKs were clustered into four major groups based on tree topology (group I, group II, group III, group IV), which agreed with the classification of *Arabidopsis* CPKs (Cheng et al., 2002). In the phylogenetic tree of five species, the *CPK* genes of five *Brassica* species and their orthologs in *Arabidopsis* formed distinct clades. Among four groups, group I contained the most *CPK* genes, including 15 *BraCPKs*, 19 *BniCPKs*, 19 *BolCPKs*, 31 *BjuCPKs*, and 37 *BnaCPKs*. A clade including *AT5G23580.1*(*CPK12*) in group I contributed the largest number of orthologs from *Brassica* species, reaching up to 36. The second largest group was group III, including 105 *CPK* genes, in which the top two clades were AT2G31500.1 (CPK24) and AT3G51850.1 (CPK13), possessing 20 and 19 members from five species respectively. There were 99 *CPK* genes in group II, and four clades, namely AT5G12180.1 (CPK17), AT5G19360.1 (CPK34), and AT4G04710.1 (CPK22), AT4G23650.1 (CPK3) occupied about 60% of orthologs from *Brassica* species in this group, reaching up to 59 genes. The group with a minimum number of members was group IV, including only three clades with a total of 32 genes. In group IV, clades AT5g66210.2 (CPK28), AT2G17890.1 (CPK16), and AT4G36070.2 (CPK18) possessed 14, 11, and 7 members respectively. In addition, some clades had very few members, such as AT4G04700.1 (CPK27), AT4G04695.1 (CPK31), AT2G35890.1 (CPK25), and AT1G50700.1 (CPK33) merely having one or two orthologs from *Brassica* species.

### Analysis of conserved motifs in BraCPK, BolCPK, and BnaCPK

**Figure 4:**
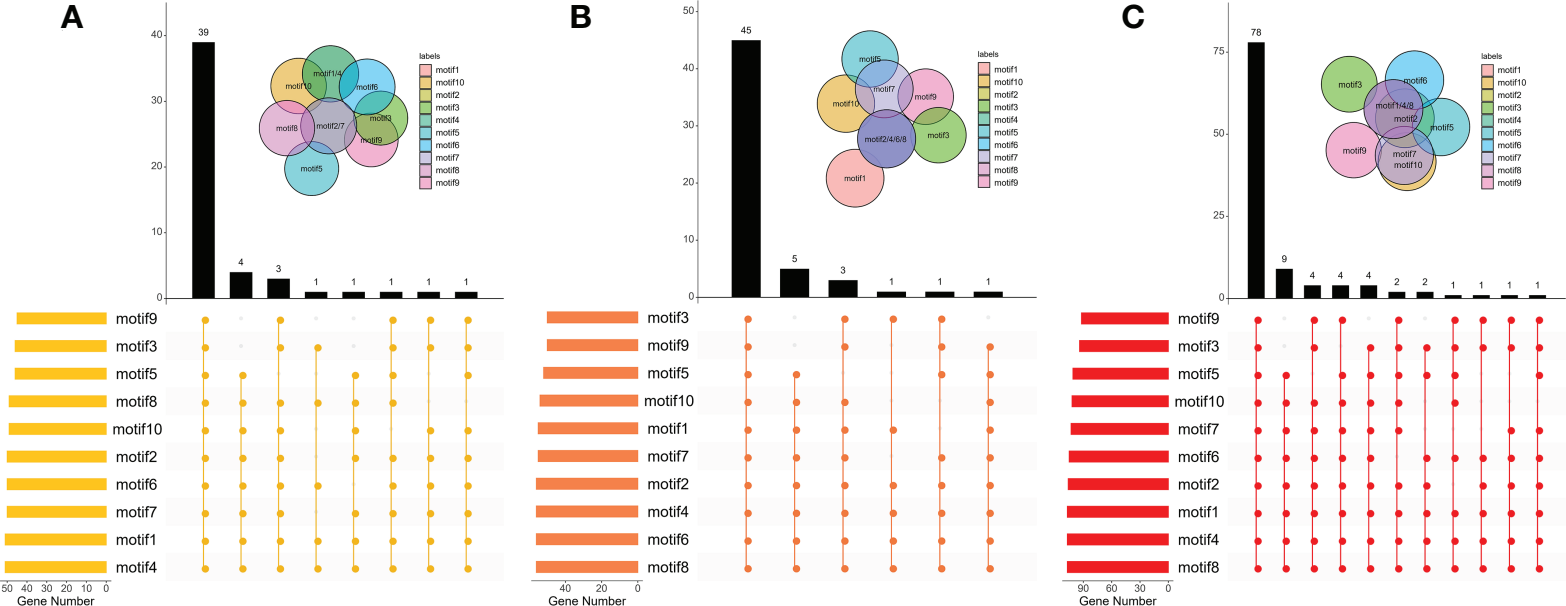
Distributions of motifs in BraCPKs **(A)**, BolCPKs **(B)**, and BnaCPKs **(C)**. The Upset plot shows the distribution of motifs in the CPKs. The number chart above represents the number of genes contained in each type of CPK. The bar chart at the bottom left represents the number of genes included in each type of motif. The dotted line shows the types of motifs contained in the group.

It was found that some CPKs duplicated or lacked certain types of motifs. Most CPKs consisted of 9 to 11 motifs, and covered all motif types (**Table S1**; **Figures S3**, **S4**). As shown in **Figure 4**, 39 of 51 CPKs in *B. rapa*, 45 of 56 in *B. oleracea*, and 78 of 107 in *B. napus* include ten types of motifs, the proportions of which were up to 76.5%, 80.4%, and 72.90%, respectively. The top three types of motifs were motif 4/1/7 in *B. rapa*, motif 8/6/4 in *B. oleracea*, and motif 8/4/1 in *B. napus*. Further analysis revealed that the composition and the number of conserved motifs or domains varied to a large extent in some CPKs (**Figure S3**). For example, BnaA02g32830D, BnaA03g46140D, BnaA05g11330D, and BraA09g025610 lacked three to five types of motifs,while BnaC09g22660D had the highest motif number of 15. In addition, some motif types in BraA02g042460 were tripled. Compared to BraCPKs and BolCPKs, BnaCPK proteins showed more diverse combinations of the motifs.

### Analysis of cis-acting elements in the promoter region of *BraCPKs*, *BolCPKs*, and *BnaCPKs*

**Figure 5:**
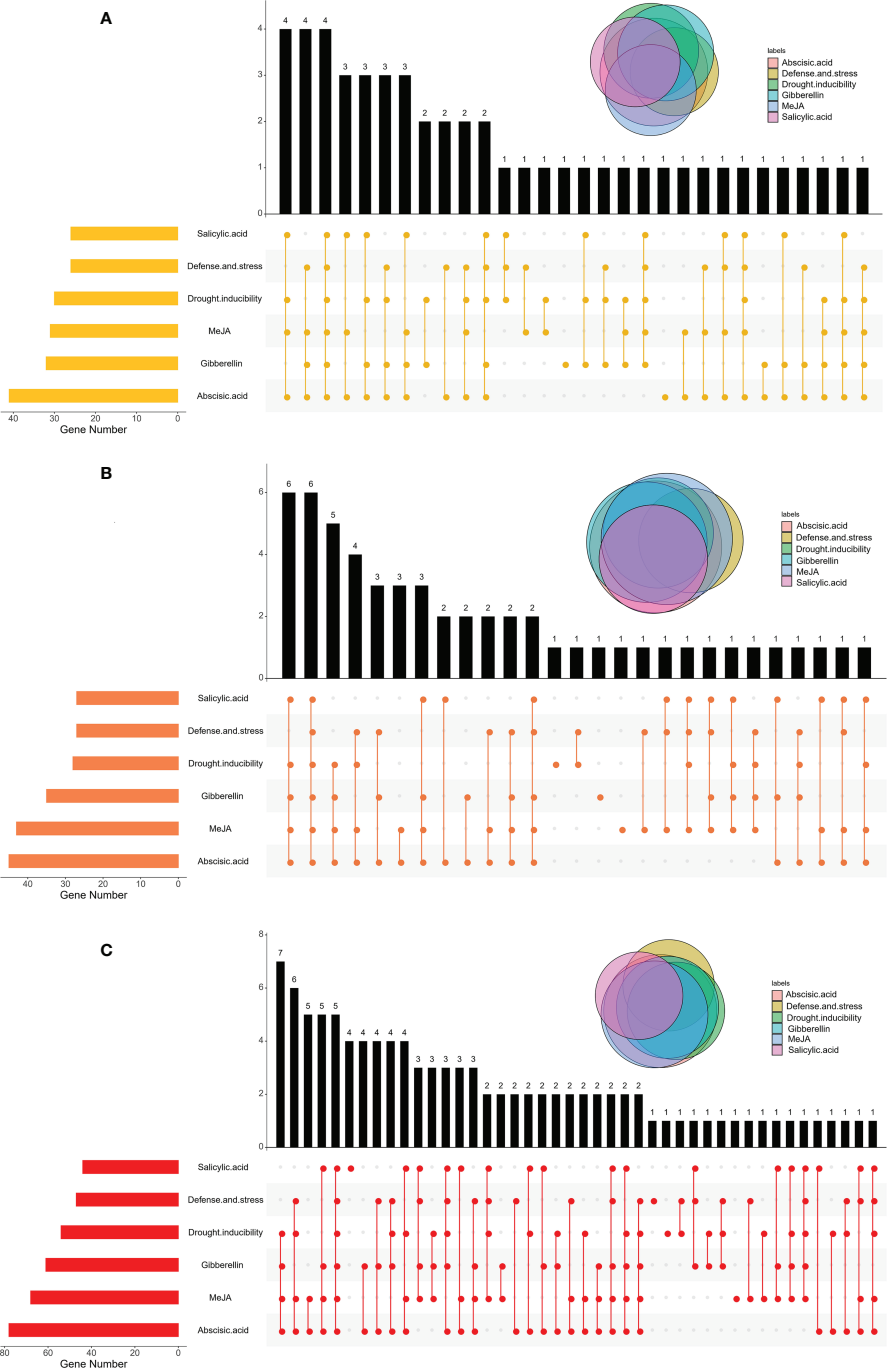
Distributions of cis-acting elements in BraCPKs **(A)**, BolCPKs **(B)**, and BnaCPKs **(C)** promoter. The Upset plot shows the distribution of cis-acting elements in CPKs promoter. The bar chart above represents the number of genes contained in each type of CPK. The bar chart at the bottom left represents the number of genes included in each type of cis-acting element. The dotted line shows the types of cis-acting elements contained in the group.

Cis-acting elements distributed in the promoter region can help predict the function of candidate genes. As most *CPK* genes are related to stress response, we further analyzed the cis-acting elements involved in stress response. Specifically, cis-acting elements are classified as MeJA responsive, gibberellin responsive, abscisic acid responsive, drought inducibility, salicylic acid responsive, and defense and stress responsive related (**Table S1**; **Figure 5** and **Figure S5**). Among them, *BnaCPKs*, *BraCPKs*, and *BolCPKs* contained the largest number of cis-acting elements related to abscisic acid responsivity, followed by MeJA or gibberellin responsivity (**Figure 5**). Most genes had three or more promoter elements, but a few genes possessed only one type of cis-acting element, such as six *BnaCPK* genes (*BnaA02g32840D, BnaA03g31330D, BnaA05g11330D, BnaA05g28200D, BnaC05g42370D* and *BnaC09g04790D*), two *BraCPK* genes (*BraA06g030040* and *BraA09g009710*), and three *BolCPK* genes (*BolC03g031480, BolC06g047200* and *BolC07g044910*) (**Figure S5**), and this presence of single type may give them corresponding functional specificities. In addition, some *CPK* genes contained no promoter elements, which may have a certain impact on gene expressions, such as *BnaA04g24150D, BnaC02g41590D*, and *BolC02g059710*, which may not be involved in plants responding to stress.

### Expression pattern of *CPK* genes induced *by Plasmodiophora brassicae*

**Figure 6:**
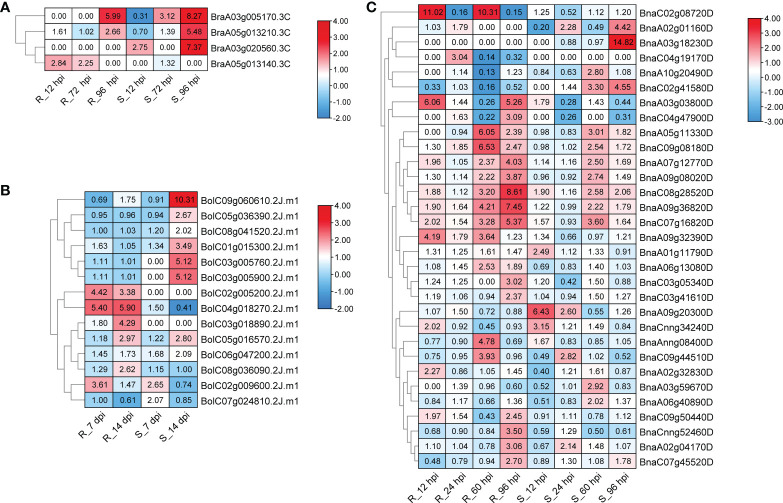
Heat maps of upregulated *BraCPKs* **(A)** *, BolCPKs* **(B)**, and *BnaCPKs* **(C)** elicited by *P. brassicae*. R and S, resistant and susceptible genotype, respectively. hpi: hours post-inoculation, dpi: days post-inoculation. The red color indicates up-regulated, and numbers in boxes indicate fold change. The legends show log_2_ fold-change. TPM value was used for heat map construction.

**Figure 7:**
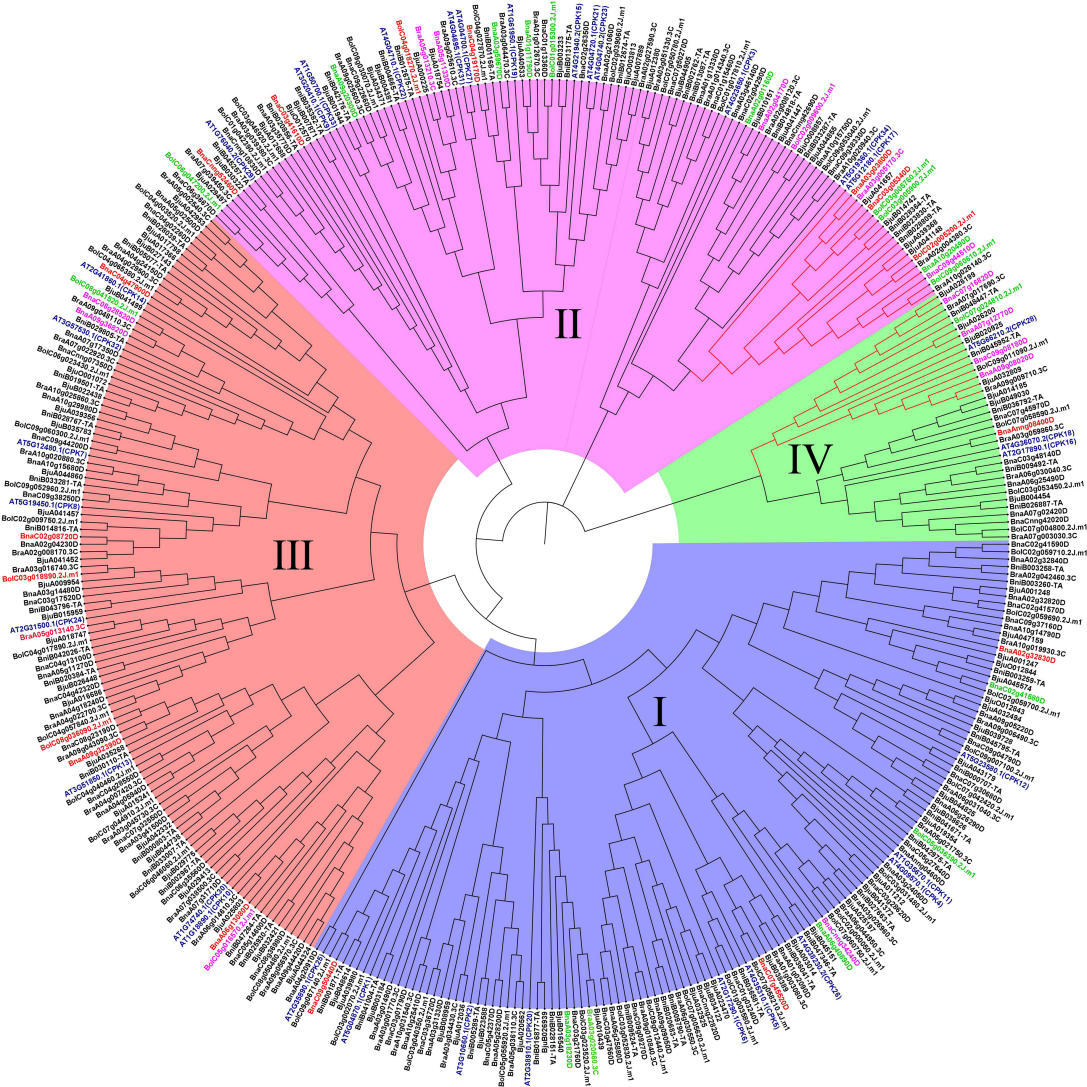
Phylogenetic tree of CPK proteins of *A. thaliana* and five *Brassica* species. The phylogenetic tree was generated by the NJ method with bootstrap analysis (2,000 bootstrap replicates) by the MEGA 11.0. The CPK proteins were clustered into four groups (group I, group I, group III, and group IV). Blue mark *AthCPK* genes, red mark upregulated-CPKs in resistance (R) genotype only, green mark upregulated-CPKs in susceptible (S) genotype only, pink mark upregulated-CPKs both resistance (R) and susceptible (S) genotypes. Red lines mark At5G12180.1 (CPK17) and AT5G66210.3 (CPK28) clades.

*Plasmodiophora brassicae* Wor., an obligate and biotrophic pathogen Rhizaria (Schwelm et al., 2015), can infect over 3,700 species in *Brassicaceae* (Hwang et al., 2012), and lead to club-root, causing significant economic losses every year (Dixon, 2009). To further reveal the roles of *CPKs* in *P. brassicae* defense responses, we analyzed the corresponding expression profile data available online. Three RNA-seq datasets were downloaded and analyzed (Chen et al., 2015; Zhang et al., 2016; Li et al., 2019). In total, 43 of 51 *BraCPK*, 49 of 56 *BolCPK*, and 100 of 107 *BnaCPK* genes showed expressional fluctuation after inoculation in resistant (R) and susceptible (S) plants (**Figure S6**). The expression of most *CPK* genes were down-regulated, and only 4 *BraCPK*, 14 *BolCPK* and 31 *BnaCPK* genes were induced by *P. brassicae* in resistant (R) or susceptible (S) materials at a certain time point (**Figure 6**). Among them, most *CPK*s presented expression specificity between R and S, such as 18 *CPK*s that were induced merely in R, including 13 *BnaCPK*s (*BnaAnng08400D*, *BnaC03g05340D*, *BnaA03g03800D*, *BnaC04g19170D*, *BnaC03g41610D*, *BnaCnng52460D*, *BnaC03g47900D*, *BnaC02g08720D*, *BnaA09g32390D*, *BnaA06g13080D*, *BnaA02g32830D*, *BnaC07g45520D*, *BnaC09g5044D*), one *BraCPK* (*BraA05g013140.3C*), and four *BolCPKs* (*BolC02g005200.2J.m1*, *BolC04g018270.2J.m1*, *BolC03g018890.2J.m1*, *BolC08g036090.2J.m1*). Additionally, 17 *CPK*s were only induced in S, including eight *BnaCPK*s (*BnaA10g20490D*, *BnaA02g01160D*, *BnaA01g11790D*, *BnaA03g59670D*, *BnaA09g20300D*, *BnaC02g41580D*, *BnaA06g40890D*, *BnaA03g18230D*), one *BraCPK* (*BraA03g020560.3C*), and eight *BolCPKs* (*BolC07g024810.2J.m1*, *BolC09g060610.2J.m1*, *BolC03g005900.2J.m1*, *BolC03g005760.2J.m1*, *BolC01g015300.2J.m1*, *BolC06g047200.2J.m1*, *BolC08g041520.2J.m1*, *BolC05g036390.2J.m1*). Some *CPKs* were highly induced reaching a level of 14.82 (*BnaA03g18230D*, 96 hpi in S), 11.02/10.31 (*BnaC02g08720D*, 12 hpi/60 hpi in R), and 10.31 (*BolC09g060610.2J.m1*, 14 dpi in S) times comparing to their initial concentrations. Apart from *BnaC02g08720D*, other R-specific induced genes, like *BnaA03g03800D* (6.06 folds in 12 hpi, 5.26 folds in 96 hpi) and *BolC04g018270.2J.m1* (5.4 folds in 7 dpi, 5.9 folds in 14 dpi) might also be good candidates for *P. brassicae* resistance research. Moreover, in the phylogenetic tree built by CPKs from *Arabidopsis* and five *Brassica* species, up-regulated genes were distributed in all groups (**Figure 7**). It is remarkable that the majority of *BraCPK*, *BolCPK*, and *BnaCPK* genes in two families, orthologs of AT5G66210.2 (CPK28) and AT5G12180.1 (CPK17), were up-regulated after *P. brassicae* inoculation, underlining their functional conservation during evolution.

## Discussion

*B. napus* (AACC, 2n = 38) and *B. juncea* (AABB, 2n = 36) are both allopolypoid species formed by the hybridization of two diploid species. *B.napus* is formed through the hybridrization of *B.rapa* (AA = 20) and *B.oleracea* (CC = 18), while the formation of *B. juncea* occurs through the hybridization of *B.rapa* (AA = 20) and *B. nigra* (BB = 16) (Chalhoub et al., 2014; Lu et al., 2019). In our phylogenetic tree, the division of groups was consistent with that of *A. thaliana* (Cheng et al., 2002). The evolution of *CPK* genes was relatively conserved among the five *Brassica* species. In general, the gene number of *BraCPKs* was almost equal to that of *BolCPKs* and *BniCPKs* in the same clade, with duplicated number for *BnaCPKs* and *BjuCPKs.* Nevertheless, in terms of individual branches, the quantity of *BjuCPK* genes was less than two times of that of *BraCPKs* and *BniCPKs*, which might be due to inaccurate assembly occurring during genetic recombination or imperfect sequencing. When selecting *Arabidopsis CPKs* as a reference, there was no doubt that the gene expansion occurred among five species during evolution, especially in some clades, such as Clade AT5G23580.1(CPK12) and AT2G31500.1(CPK24). Of course, the possibility that certain *Arabidopsis CPK* genes have been lost during evolution cannot be ruled out. As the structure of exons/introns and the type and number of introns can account for the evolutionary history of organisms, we analyzed the exon/introns substructure of all *CPKs* in this study. Genes in group IV had the most exons, from eleven to thirteen, not including BjuA026200. Most of the *CPK* genes in other groups contained less than ten exons. In general, *CPK* genes clustered in the same subfamily showed similar exon/intron structures, indicating a close evolutionary relationship between them.

At present, *CPK* genes have been identified in *Arabidopsis* (Cheng et al., 2002), rice (Asano et al., 2005), soybean (Liu et al., 2016), maize (Kong et al., 2013; Ma et al., 2013), cucumber (Xu et al., 2015), tomato (Hu et al., 2016) and many other plants (Yang et al., 2017; Wen et al., 2020; Zhang et al., 2020; Zhao et al., 2021), and their roles in biotic and abiotic stresses have been understood to some extent (Asano et al., 2012; Boudsocq and Sheen, 2013; Ranty et al., 2016; Bredow and Monaghan, 2019). Previous studies found that plant CPKs are involved in abscisic acid signaling pathways (Liu et al., 2006, 1; Yu et al., 2006; Zhu et al., 2007; Jiang et al., 2013; Chen et al., 2019), jasmonic acid signaling pathway (Ulloa et al., 2002; Hettenhausen et al., 2013; Matschi et al., 2015), gibberellin signaling pathway (Abo-el-Saad and Wu, 1995; Abbasi et al., 2004; Ishida et al., 2008; Nakata et al., 2009), and also response to drought stress (Ma and Wu, 2007; Cieśla et al., 2016, 2; Bundó and Coca, 2017; Huang et al., 2018). In line with these results, a large number of cis-acting elements related to abscisic acid, jasmonic acid, gibberellin, and drought stress response were found in the promoter region of *Brassica CPK* genes in this study.

*Plasmodiophora*, as one class of obligate biotrophic pathogens (Ludwig-Müller et al., 2009), is highly destructive to many *Brassica* plants and exerts negative effect on their quality and yield (Dixon, 2009), which seriously hinders the development of agricultural industry. In this study, utilizing the expression data from the resistant (R) and susceptible (S) lines in three *Brassica* species (*B. rapa*, *B. oleracea*, and *B. napus*), we found 49 up-regulated genes induced by *P. brassicae*. These up-regulated genes would be helpful to identify positive regulators in the process of response to *P. brassicae* (**Figure 6**). Interestingly, the distribution of these 49 genes was extremely uneven among the three *Brassica* species. Among them, 31 genes were found in *B. napus*, accounting for 29% of *BnaCPKs*, while only 4 genes were found in *B. rapa*, with a proportion of 8%, suggesting that BnaCPKs played an important role in *B. napus* taking charge in response to *P. brassicae* infection, while BraCPKs might be mainly involved in other stresses. Moreover, of the 49 up-regulated genes, 18 genes were specifically up-regulated in R, and those genes identified from incompatible interactions might be good candidates for further functional assignment of *CPK* genes against *P.brassicae* infection. The up-regulated genes of the three species were distributed in the four groups in varying amounts (**Figure 7**). There were 9 *CPK* genes in group I, 23 in group I, 11 in group III, and only 6 in group IV, suggesting that *CPK* genes involved in *P.brassicae* response evolved independently in four groups along with the functional divergence in *Brassica* species. However, the genes in group I may play a broader role in response to *P.brassicae* infection. It is worth noting that more genes in clade AT5G12180.1 (CPK17) and clade AT5G6621.2 (CPK28) were up-regulated by *P.brassicae* induction. Evolutionarily, these two clades of genes were functionally conserved, and most likely to be active gene clusters during response to *P.brassicae* infection in *Brassica* species.

## Conclusion

The gene and protein characterization, localization, evolution, and expression analysis of highly conserved *CPK* genes in *Brassica* showed that most of the *CPK* gene families in this study were relatively conserved during evolution. Genome-wide identification and expression analysis of *CPK* family members can provide an alternative strategy for enhancing resistance to *P. brassicae* in *Brassica* species. These identified genes may be good candidates for further identification of *CPK* genes of *P. brassicae* infection and provide theoretical basis and preliminary guidance for prevention of *P. brassicae* pathogen damage to *Brassica* plants.

## Data availability statement

The original contributions presented in the study are included in the article/**Supplementary Material**. Further inquiries can be directed to the corresponding authors.

## Author contributions

JL: formal analysis, writing the original draft, data curation, and validation. NY: investigation, resources, and visualization. YZ: data curation. ZC: data validation. TZ: writing-review and editing, and supervision. WL: conceptualization, writing-review and editing, and supervision. All authors contributed to the article and approved the submitted version. All authors have read and agreed to the published version of the manuscript.

## Funding

This work was supported by grants from the Chongqing Municipal Education Commission (KJQN201900533) to JL, the National Natural Science Foundation of China (31171588) and the Chongqing Municipal Education Commission (KJZD-K202200508) to TZ, and Chongqing Bureau of Human Resources and Social Security Postdoctoral Funding (0019) to WL. JL and WL were partly supported by fellowships from China Scholarship Council (CSC).

## Acknowledgments

We sincerely thank Athena Li from the University of British Columbia for the critical reading of the manuscript.

## Conflict of interest

The authors declare that the research was conducted in the absence of any commercial or financial relationships that could be construed as a potential conflict of interest.

## Publisher’s note

All claims expressed in this article are solely those of the authors and do not necessarily represent those of their affiliated organizations, or those of the publisher, the editors and the reviewers. Any product that may be evaluated in this article, or claim that may be made by its manufacturer, is not guaranteed or endorsed by the publisher.

## Supplementary material

Supplementary Figure 1 Gene structures of CPK genes in five *Brassica* species: (A) *BraCPK*, (B) *BniCPK*, (C)*BolCPK*, (D)*BjuCPK*, (E)*BnaCPK* genes. The green bars represent CDS, the black lines represent intron, and the yellow bar represents UTR.

Supplementary Figure 2 The conserved domains of CPK proteins in five *Brassica* species. **(A)**BraCPKs, **(B)**BniCPKs, **(C)** BolCPKs, **(D)**BjuCPKs, **(E)**BnaCPKs. The green bar represents protein kinase domain (Pkinase), and the yellow bar represents EF-hand domain (EFh).

Supplementary Figure 3 The motifs in CPK proteins of *B. rapa* **(A)**, *B. oleracea* **(B)**, and *B. napus* **(C)**. Motif labeled as motif 1 - motif 10.

Supplementary Figure 4 The sequence of motif 1 - motif 10 of *B. rapa, B. oleracea*, and *B. napus.*

Supplementary Figure 5 The cis-acting elements in CPKs promoter of *B. rapa* (A)*, B. oleracea* (B), and *B. napus* (C). Approximately 2,000-bp upstream flanking fragments of the CPK genes were derived, and PlantCARE was used to predict promoter cis-elements.

Supplementary Figure 6 The expression heat map of CPK genes in *B. rapa* **(A)** *, B. oleracea* **(B)**, and *B. napus* **(C)** elicited by *P. brassicae*. R and S, resistant and susceptible genotypes, respectively. hpi: hours post-inoculation, dpi: days post-inoculation. The red color indicates up-regulated, and blue color indicates down-regulated. The legends show log_2_ fold-change. TPM value was used for heat map construction.
