# Supplementary figures and images for: Genome-wide survey of Calcium-Dependent Protein Kinases (CPKs) in five Brassica species and identification of CPKs induced by Plasmodiophora brassicae in B. rapa, B. oleracea, and B. napus
Source: Front Plant Sci. 2022 Nov 21;13:1067723. doi: 10.3389/fpls.2022.1067723 (PMC9720142; doi:10.3389/fpls.2022.1067723)

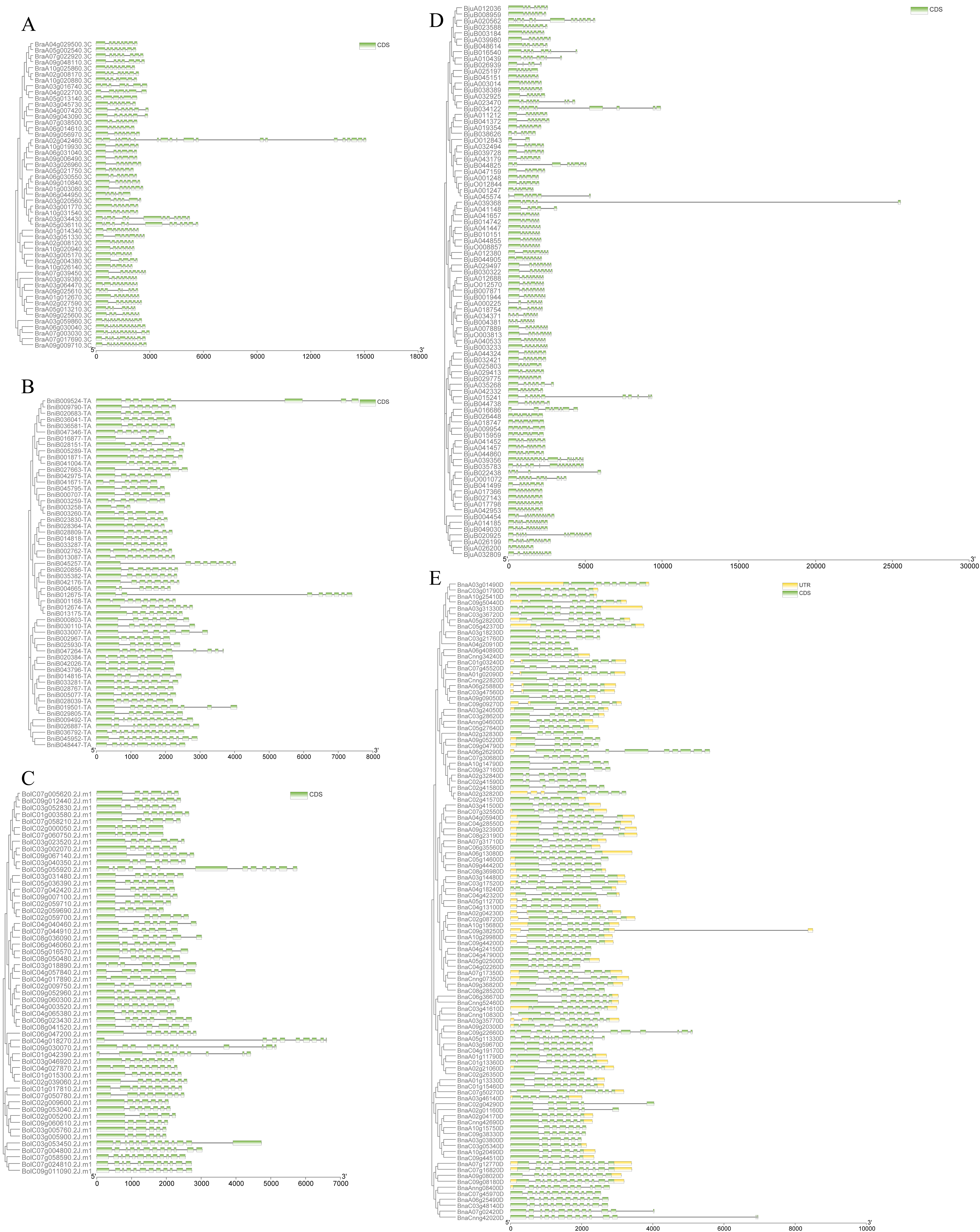

Supplement: Supplementary Figure 1 — Gene structures of CPK genes in five Brassica species: (A) BraCPK, (B) BniCPK, (C)BolCPK, (D)BjuCPK, (E)BnaCPK genes. The green bars represent CDS, the black lines represent intron, and the yellow bar represents UTR. [file Image_1.jpeg]

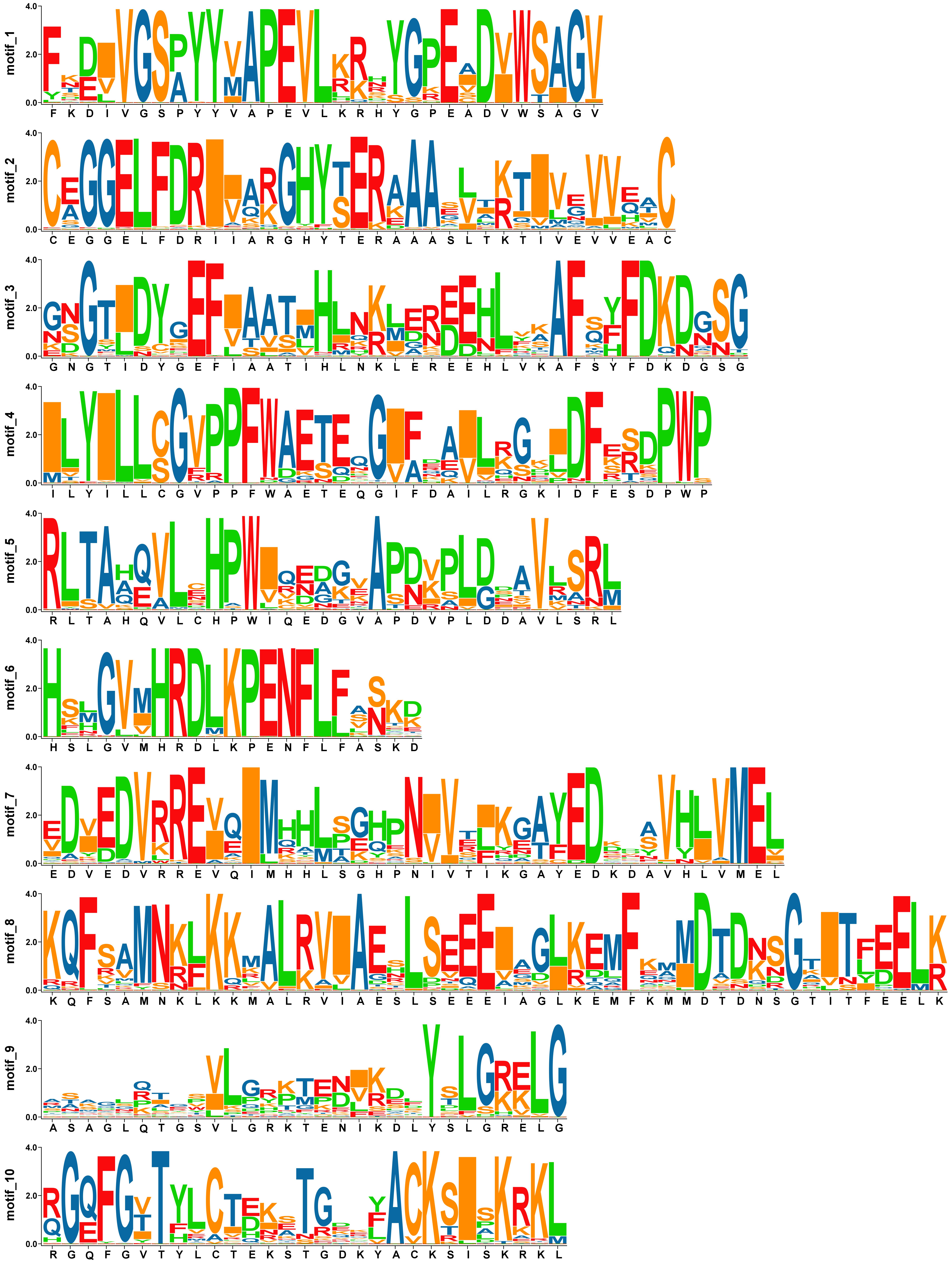

Supplement: Supplementary Figure 4 — The sequence of motif 1 - motif 10 of B. rapa, B. oleracea, and B. napus. [file Image_4.jpeg]

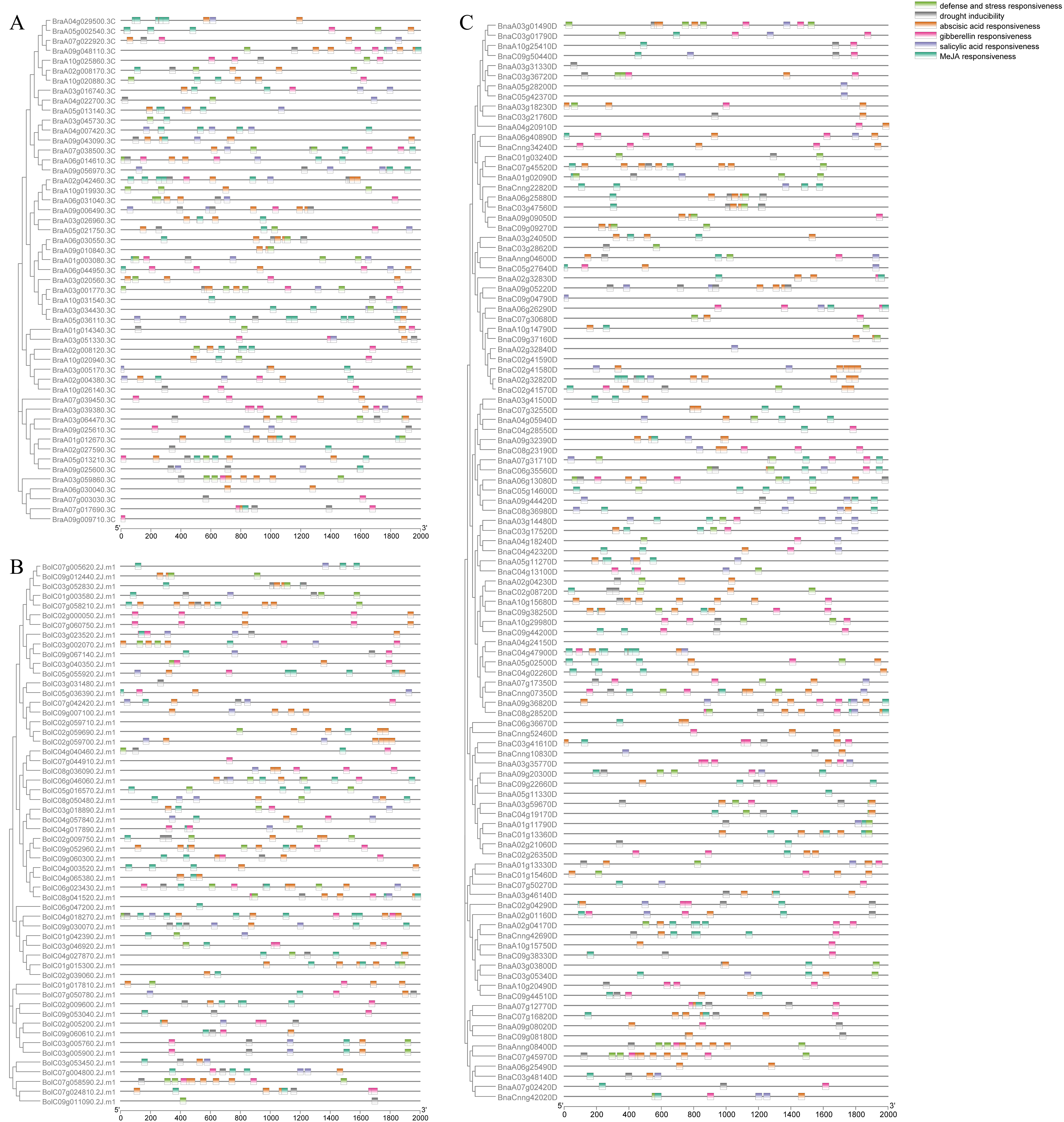

Supplement: Supplementary Figure 5 — The cis-acting elements in CPKs promoter of B. rapa (A), B. oleracea (B), and B. napus (C). Approximately 2,000-bp upstream flanking fragments of the CPK genes were derived, and PlantCARE was used to predict promoter cis-elements. [file Image_5.jpeg]
